# Diaphragmatic Point-of-Care Ultrasound in COVID-19 Patients in the Emergency Department—A Proof-of-Concept Study

**DOI:** 10.3390/jcm10225291

**Published:** 2021-11-14

**Authors:** Emanuele Pivetta, Irene Cara, Giulia Paglietta, Virginia Scategni, Giulia Labarile, Maria Tizzani, Giulio Porrino, Stefania Locatelli, Gilberto Calzolari, Fulvio Morello, Milena Maria Maule, Enrico Lupia

**Affiliations:** 1 Division of Emergency Medicine and High Dependency Unit, Città della Salute e della Scienza di Torino University Hospital, Corso Bramante 88, 10126 Turin, Italy; mariatizzani@yahoo.it (M.T.); porgi74@yahoo.it (G.P.); stefylocatelli@libero.it (S.L.); gilberto.calzolari83@gmail.com (G.C.); fulvio.morello@unito.it (F.M.); enrico.lupia@unito.it (E.L.); 2 Residency Program in Emergency Medicine, University of Turin, Via Verdi 8, 10124 Turin, Italy; irene.cara@edu.unito.it (I.C.); giulia.paglietta@unito.it (G.P.); virginia.scategni@gmail.com (V.S.); labarile.giulia@gmail.com (G.L.); 3 Department of Medical Sciences, University of Turin, Corso Dogliotti 14, 10126 Turin, Italy; milena.maule@unito.it; 4 Cancer Epidemiology Unit and CPO Piemonte, Città della Salute e della Scienza di Torino University Hospital, Via Santena 7, 10126 Turin, Italy

**Keywords:** point-of-care ultrasound, diaphragm, COVID-19

## Abstract

Background: Lung Ultrasound Evaluation (LUS) is usefully applied in the Emergency Department (ED) to patients with suspected or confirmed COVID-19. Diaphragmatic Ultrasound (DUS) may provide additional insight into ventilatory function. This proof-of-concept study aimed to evaluate the feasibility of LUS and DUS in a third level ED during the COVID-19 pandemic. Methods: Adult patients presenting with COVID-19 symptoms were eligible. After the physical examination, both LUS and DUS (i.e., diaphragmatic motion and thickness) were performed. All patients were followed after 30 days to determine their need for ventilation, admission, and/or a new ED evaluation after discharge. The diagnostic accuracies of diaphragm measurements in assessing the risk of the 30-day outcome were calculated as well as the measurements’ usefulness. Bland–Altman plots were used for comparing bedside and off-line diaphragm measurements. Results: 118 patients were enrolled. Median thickness and motion were 1.7 mm (iqr 0.4) and 1.8 cm (iqr 0.7), respectively, with a mean difference of 0.009 mm (95% CI −0.037–0.056 mm) and −0.051 cm (95% CI −0.108–0.006 cm), respectively. The 30-day outcome was associated with an increase in thickness (OR 5.84, 95% CI 0.96–35.4), and a lower motion (OR 0.49, 95% CI 0.2–1.21). Conclusion: DUS seemed to be feasible and reliable in the ED in a population of patients presenting with symptoms related to COVID-19 infection.

## 1. Introduction

The global pandemic of COVID-19 has led to wider use of and accumulating evidence for, point-of-care ultrasound (POCUS), a non-invasive tool well suited for bedside management and prevention of infection propagation [1]. Since SARS-CoV-2 infection primarily affects the respiratory system, lung ultrasound (LUS) has played a major role [2]. LUS allows clinicians to detect interstitial pneumonia and to quantify its severity in the Emergency Department (ED). The predominant pattern is of varying degrees of interstitial syndrome and alveolar consolidations, which can be seen as the presence of focal, multifocal and/or confluent B-lines and of pleural irregularities [3,4]. A scoring system that classifies each pathological area in the lung based on the number of sonographic artifacts (e.g., 0 is a normal lung, 3 is the pneumonia pattern) is used to quantify the severity of the pulmonary impairment detected by LUS [3,5,6].

Diaphragm weakness and paralysis have already been described as contributors to respiratory symptoms, and sonographic features of the diaphragm have already been assessed in some settings. The diaphragm ultrasonography (DUS) assesses three different measurements: thickness, thickening fraction, and motion (also reported as excursion). 

The thickness and the motion are the muscular length and its respiratory excursion, respectively, and they are measured using the linear and the sector probe, respectively.

The diaphragm thickening fraction (DTF) reflects variation in the thickness during respiratory effort and is calculated as (thickness at end-inspiration—thickness at the end-expiration)/thickness at the end of the expiration [7,8,9]. The role of DUS in critical care is already demonstrated in (1) predicting the success of mechanical ventilation weaning, (2) assessing the progression of atrophy in mechanically ventilated patients and in performing protective ventilation, (3) evaluating possible damages after surgery [7,8,9]. On the other hand, little data can be found regarding the usefulness of DUS in the ED for the evaluation of patients with acute respiratory failure, especially among patients affected by COVID-19. A pilot study from Corradi and co-authors was previously performed in order to evaluate the role of DUS in 27 patients with COVID-19 pneumonia who underwent non-invasive ventilation; DTF was identified as a predictor of CPAP failure, as its values inversely correlate with its success (i.e., the lower the DTF values, the more likely the CPAP failure) [10]. 

This is a proof-of-concept study designed to evaluate the feasibility of LUS and DUS evaluation in a third-level ED in Italy, during the COVID-19 pandemic. 

## 2. Materials and Methods

This study was conducted at the ED of the Città della Salute e della Scienza di Torino, University Hospital, Turin, Italy.

We enrolled adult patients presenting to the ED for acute symptoms commonly associated with SARS-CoV-2 infection (e.g., fever, cough, dyspnea, ageusia, anosmia, asthenia, myalgia), regardless of the need for hospitalization. 

Data on past medical history and acute symptoms were collected along with vital signs, laboratory tests, and arterial blood gas analysis results. 

After the physical examination, both LUS and DUS were performed. We used cart-based sonographic machines (Esaote Mylab5 and Esaote Mylab7, Genova, Italy) and a handheld device (Butterfly IQ; Butterfly Network Inc., Guiford, CT, USA).

LUS was performed using a 12-point scanning protocol, identifying three lung fields for each hemithorax (anterior, lateral, and posterior) further divided into upper and lower zones. The anatomic landmarks used to determine these fields were the midclavicular line, anterior and posterior axillary lines, and mid scapular line. 

As suggested in the international literature, we assigned the LUS score to each area using a semiquantitative scoring system based on four different grades with regard to aeration of the lung: 0 for normal aeration (=presence of A-lines and up to 2 B-lines), 1 for moderate loss of aeration (= multiple single B-lines), 2 for severe loss of aeration (=multiple coalescent B-lines), and 3 for complete loss of aeration (=tissue-like pattern, consolidation, air bronchograms) [1,2,11,12]. 

The physician who performed the ultrasound evaluation also registered short videos (5–7 s), so that the LUS score could be calculated while collecting data for the study. DUS was tested by evaluating both the thickness and the motion and taking three measures of both of them. We visualized the right side of the diaphragm, exploiting the acoustic window made by the liver.

For each patient, three measurements of the diaphragm end-expiratory thickness were measured at the apposition zone (the region where the muscle is adjacent to the lower rib cage), positioning the transducer in a cranio-caudal direction on the anterior axillary line between the 7th and the 8th or between the 8th and the 9th rib.

**Figure 1:**
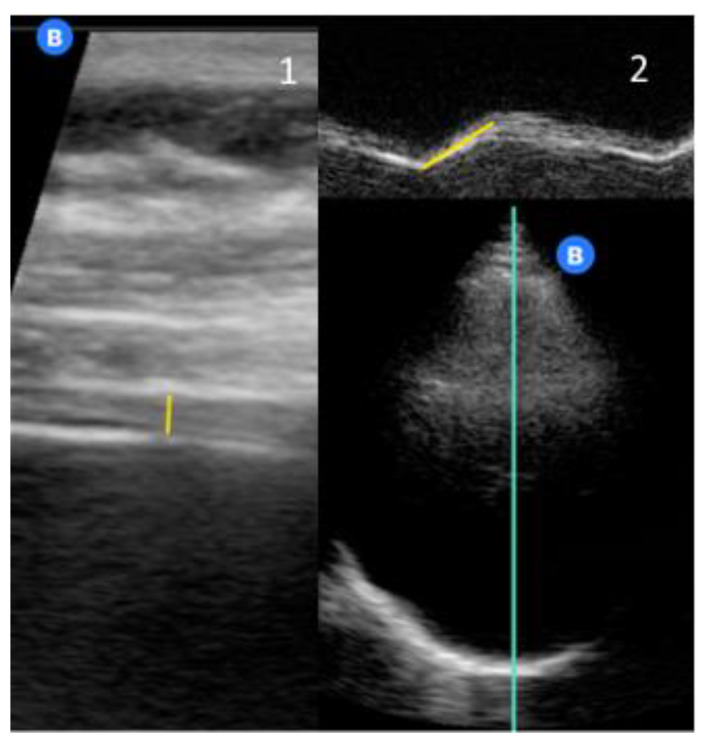
Example of DUS measurements: Panel 1 shows diaphragmatic thickness at end-expiration; Panel 2 shows diaphragmatic motion (the white B in the blue dot is the probe marker).

We used a high-frequency probe or a musculoskeletal preset as previously reported in the international literature [7,8,9,13] (Figure 1). At a depth of 1.5–3 cm, a three-layered structure can be identified, two parallel echogenic layers (the parietal pleura and the peritoneum) and, between them, the diaphragm, seen as a less echogenic structure (i.e., diaphragmatic thickness).

Thickness was measured perpendicularly to the direction of the muscular fibers, excluding the pleural and peritoneal membrane. 

Diaphragmatic motion (or excursion) was measured in M-mode, using a cardiac probe (or a cardiac ultrasound preset) positioned below the costal arch at the midclavicular line, and angling the ultrasound beam cranially and posteriorly (Figure 1) [7,8,13,14].

Thirty days after the first ED evaluation, the outcome was assessed for all enrolled patients. Discharged patients were telephonically followed up, assessing the need for a new ED visit. For patients admitted to the hospital from the ED, the patient chart was evaluated by the authors (I.C., G.P., and E.P.), assessing admission outcomes and the need for non-invasive and/or invasive mechanical ventilation in the same period of time. 

Characteristics of patients are presented as proportions for categorical variables and means with standard deviations (SD) or medians with interquartile ranges (IQR) for continuous variables, as appropriate.

The diagnostic accuracies of diaphragm measurements in assessing the risk of the 30-day outcome were calculated through sensitivity, specificity, positive predictive values and negative predictive values, positive and negative likelihood ratios, and the area under the receiver operating characteristic curve [15]. Net benefits and decision curves were used to evaluate the measurements’ usefulness [16].

Bland–Altman plots were used for comparing bedside and off-line diaphragm measurements as blindly re-assessed by an expert operator (E. P.) [17]. 

Images were recorded at the bedside using a Butterfly iQ (Butterfly Network Inc - linear preset, 10–5 MHz), and Esaote mylab5 and mylab7 equipped with linear probes (12–8 MHz). All images were collected in mp4 and DICOM format. Along with diaphragm examination, a lung assessment with an LUS score was performed and stored. 

Furthermore, if it was a proof-of-concept study, we estimated power of 0.9 for a cohort of 100 patients with thickness examined bedside and offline, an alpha of 0.05, and a mean difference of 0.3 (SD 1).

The study was approved by the Hospital institutional review board (N. CS3/23). All patients or their substitute decisionmakers provided informed consent. The study was conducted in accordance with the principles of the Declaration of Helsinki for clinical research involving human subjects.

## 3. Results

Between December 2020 and January 2021, 118 patients were enrolled (51, 43.2%, were female) with a median age of 67.5 years (interquartile range, iqr, 26.2). Among them, 109 patients had a positive swab for SARS-CoV-2 infection, and 27 were discharged from the ED. Twenty-six patients experienced a 30-day outcome (including death in the ED or during the admission, need for non-invasive ventilation, and a further ED examination after discharge). Table 1 summarizes patient comorbidities, symptoms, and vital parameters as reported during the ED evaluation.

One hundred and nine patients received three diaphragm thickness measurements, and 97 received three motion evaluations. The LUS score was 2 (iqr 5), 8 (iqr 9), and 12.5 (iqr 6.5) for discharged, admitted, and deceased patients, respectively (*p* < 0.01). 

Median diaphragm thickness and motion were 1.7 mm (iqr 0.4) and 1.8 cm (iqr 0.7), respectively. Thickness was lower (1.66 mm, iqr 0.37, vs 1.83 mm, iqr 0.43, p 0.03) and motion was higher (1.8 m, iqr 0.68, vs 1.66, iqr 1, p 0.27) in patients who experienced a 30-day outcome than in patients without events. 

**Figure 2:**
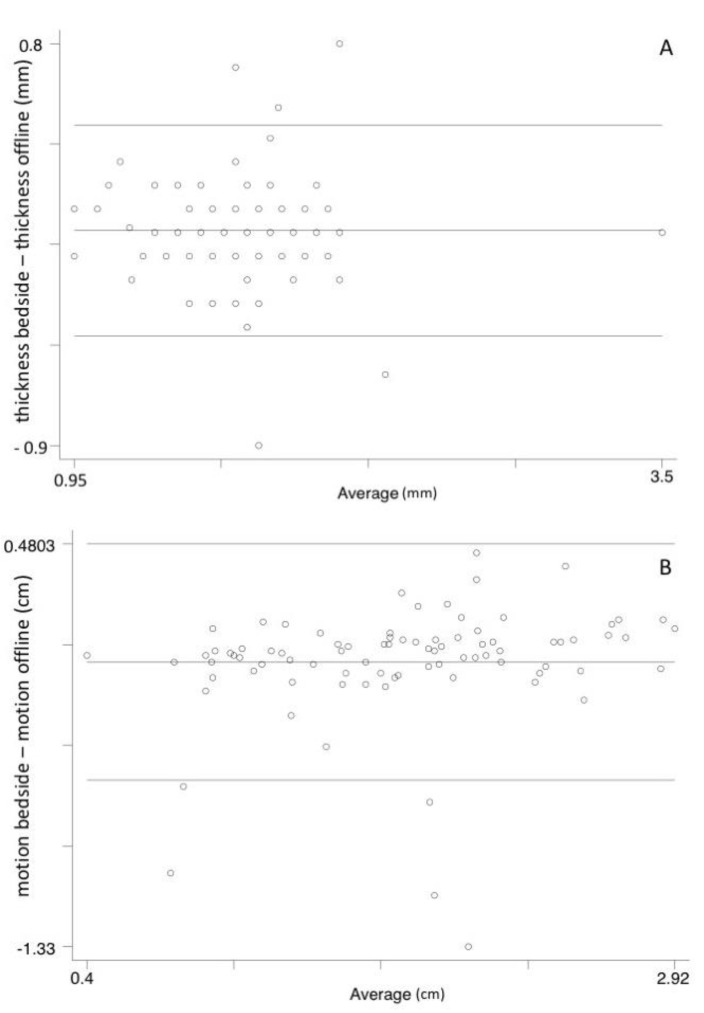
Bland–Altman plots for agreement between diaphragm thickness and motion measurement bedside and offline (Panel (**A**) and (**B**), respectively).

Figure 2 shows Bland–Altman plots for the comparison between bedside and offline thickness and motion. The mean difference was 0.009 mm (95% CI −0.037–0.056 mm; limits of agreement −0.436–0.455 mm) and −0.051 cm (95% CI −0.108–0.006 cm; limits of agreement −0.582–0.480 cm), respectively. 

The risk of a 30-day outcome was associated with an increase in diaphragmatic thickness (OR 5.84, 95% CI 0.96–35.4), a lower motion (OR 0.49, 95% CI 0.2–1.21), and an increase in LUS score (OR 1.09, 95% CI 0.99–1.20). 

The thickness measurements showed an AUC for a 30-day outcome of 64.8% (95% CI 52–77.5%) that increased to 78.5% (95% CI 68.8–89.1%), using a model including age and LUS score. 

Motion had an inverse effect on outcome with an AUC of 57.2% (95% CI 43.7–70.7%) that became 75.1% (95% CI 64.6–85.5%) in a model including age and LUS score. 

**Figure 3:**
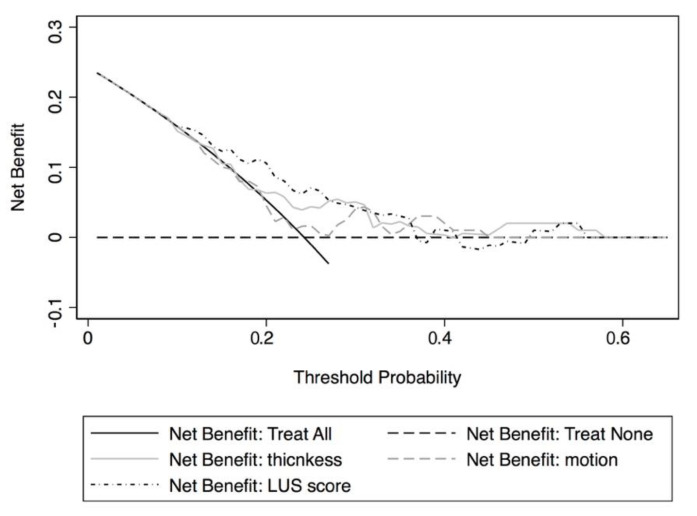
Decision curves for 30-day outcomes for diaphragmatic thickness, motion, and LUS score at ED evaluation.

Figure 3 shows the decision curve analysis for both DUS variables and LUS scores. 

## 4. Discussion

In the present study, the DUS evaluation was feasible and reliable among patients presenting in the ED during the COVID-19 pandemic. 

Two basic measurements were chosen to evaluate the feasibility of diaphragm evaluation in the ED: thickness and motion. In both cases, Bland–Altman plots (Figure 3) reported a very small variability in measurements despite the small evaluated dimensions (usual thickness at the end of expiration, in quiet breathing, is less than 3 mm and motion, in the same condition, is about 2 cm) [18]. Comparing bedside and off-line measurements, the mean difference and limits of agreement were lower than 0.1 mm for thickness, and lower than 0.1 and 0.6 cm for motion. Along with feasibility, we evaluated diagnostic accuracy and clinical utility of DUS for the prediction of 30-day outcomes. 

Both thickness and motion show a moderate accuracy, with an AUC between 75 and about 80% in models built with a few additional easy to collect independent variables (i.e., age and LUS score). Clinical usefulness was lower, with only 10% in NBs (see Figure 3) for each measurement. However, the present study was not primarily designed to evaluate these aspects and additional ad hoc studies are needed to better clarify diaphragm ultrasound accuracy and clinical usefulness. 

To our knowledge, DUS has been evaluated in only one study conducted on ED patients by Cammarota and colleagues [18]. This study enrolled COPD patients presenting to the ED for acute exacerbation subjected to NIV and monitored them at three different timings during ventilation. Among 21 patients, motion was greater among patients who experienced NIV success. Motion results were similar to those in our cohort, but the thickness was different: it was lower in our cohort than in the Cammarota paper. This difference might have several explanations. The setting of the two studies was similar (two Northwestern Italian university hospitals), but study populations were substantially different, with COPD patients presenting with hypercapnic acute respiratory failure related to COPD exacerbation or pneumonia.

Both studies underlined a first possible limitation related to the DUS. The need for evaluating small measurements could imply a large variability. Moreover, diaphragmatic evaluation might not be easy to perform for inexperienced operators.

Testa and colleagues, in 2011, showed a 4 mm difference in motion median measurements between expert and naive operators [19]. The authors assessed this difference in healthy subjects evaluated by two operators with different expertise in point-of-care ultrasound. Interobserver variability was 5.2 mm at quiet breathing. 

### Limitations

Our sample size was only 100 patients, but this is higher than most of the studies already published on DUS [20]. This limited the power to assess the prognostic value of DUS in respiratory diseases. This represents an important aspect to be addressed in the future since very few studies have evaluated this outcome [13]. 

Due to the study outcome and the setting, we chose two easy-to-collect measurements. In particular, we did not evaluate the thickening fraction [20]. This has been described in ICU studies as an informative diaphragm assessment, defined as the ratio between diaphragm the thickness measured at the end-inspiration minus the thickness at end-expiration, and the thickness at end-expiration [21]. The thickening fraction has been shown to predict weaning from mechanical ventilation in intensive care units [21,22,23,24,25,26,27] and early non-invasive ventilation failure among COPD patients [17]. 

In addition, we did not collect data on possible dysmotility patterns as reported in other cohorts [28]. 

As reported by Testa and colleagues [19], the risk of variability is present in our study also, and it represents an additional limitation. In our cohort, all emergency physicians participating in the study were trained in point-of-care ultrasound, and Bland–Altman plots showed a very low variability. These results could be due to the operators’ expertise, but additional studies are needed to clarify this point. 

## 5. Conclusions

In conclusion, DUS was feasible and reliable in the ED in a population of patients presenting with symptoms related to COVID-19 infection. Additional studies are needed to clarify the accuracy, prognostic value, and external reproducibility of DUS. 

## Figures and Tables

**Table 1:** Demographic characteristics and symptoms as reported at Emergency Department presentation.

	COVID-19 Patients (*n* = 109)
Days from symptoms onset, median (iqr)	6 (7)
Hypertension, %	49 (45.8%)
Diabetes, %	15 (13.9%)
Active smoking, %	5 (6.9%)
Chronic renal impairment, %	9 (8.3%)
Active neoplasm, %	12 (11.1%)
Coronary artery disease, %	10 (9.3%)
Stroke, %	5 (4.6%)
Dyspnea, %	51 (47.2%)
Sore throat, %	5 (4.6%)
Fever, %	71 (65.4%)
Cough, %	60 (55.6%)
Nausea and vomit, %	13 (12%)
Ageusia and anosmia, %	17 (15.7%)
Diarrhea, %	19 (17.6%)
Systolic blood pressure, mmHg, median (iqr)	130 (25)
heart rate, beat per minute, median (iqr)	90 (26.5)
Respiratory rate, breaths per minute, median (iqr)	20 (8)
partial pressure of oxygen, mmHg, median (iqr)	74 (25)
partial pressure of carbon dioxide, mmHg, median (iqr)	33 (8)
lactates, mmol/L, median (iqr)	1.3 (0.09)
